# *miR-381-3p* Inhibits Intramuscular Fat Deposition through Targeting *FABP3* by ceRNA Regulatory Network

**DOI:** 10.3390/biology11101497

**Published:** 2022-10-13

**Authors:** Yao Jiang, Jiali Liu, Huatao Liu, Wei Zhang, Xiaojin Li, Linqing Liu, Mei Zhou, Jieru Wang, Shiguang Su, Xiangdong Ding, Chonglong Wang

**Affiliations:** 1 Key Laboratory of Pig Molecular Quantitative Genetics of Anhui Academy of Agricultural Sciences, Anhui Provincial Key Laboratory of Livestock and Poultry Product Safety Engineering, Institute of Animal Husbandry and Veterinary Medicine, Anhui Academy of Agricultural Sciences, Hefei 230031, China; 2 National Animal Husbandry Service, Beijing 100125, China; 3 National Engineering Laboratory for Animal Breeding, Laboratory of Animal Genetics, Breeding and Reproduction, Ministry of Agriculture, College of Animal Science and Technology, China Agricultural University, Beijing 100193, China; 4 Key Laboratory of Animal Genetics, Breeding and Reproduction (Poultry) of Ministry of Agriculture, Institute of Animal Science, Chinese Academy of Agricultural Sciences, Beijing 100193, China

**Keywords:** intramuscular fat deposition, ceRNA, *miR-381-3p*, *FABP3*, *PPAR* signalling pathway

## Abstract

**Simple Summary:**

Intramuscular fat (IMF) deposition is an important determinant of pork quality and a complex process facilitated by non-coding ceRNAs. Emerging evidence suggests that IMF deposition is a complex process facilitated and associated with non-coding RNAs, such as microRNAs and long non-coding RNA (lncRNA). In our study, whole-transcriptome sequencing analysis was performed using longissimus dorsi samples of six low and high IMF Berkshire × Anqing Sixwhite crossbred pigs. Differentially expressed (DE) lncRNAs, miRNAs, and mRNAs, were screened and constructed 34 competing endogenous RNA (ceRNA). Following weighted gene co-expression network analysis, only one ceRNA, *lncRNA4789/miR-381-3p/FABP3*, that showed similar DE trend in longissimus dorsi tissue was retained. Furthermore, dual-luciferase reporter assays further indicated that *FABP3* was a direct, functional target of *miR-381-3p while* overexpressed *lncRNA4789* attenuated the effect of *miR-381-3p* on *FABP3* by sponging *miR-381-3p*. Cell function verification experiment demonstrated that *miR-381-3p* suppressed IMF deposition by inhibiting preadipocyte cell differentiation and lipid droplet deposition via the suppression of *FABP3* expression in the peroxisome proliferator-activated receptor signalling pathway, whereas *lncRNA4789* rescued *FABP3* expression by sponging *miR-381-3p*. Our study may aid in identifying novel molecular markers for its optimization in IMF which is of importance in breeding for improving pork quality.

**Abstract:**

Intramuscular fat (IMF) deposition is an important determinant of pork quality and a complex process facilitated by non-coding ceRNAs. In this study, 52 Berkshire × Anqing Sixwhite crossbred pigs were slaughtered to measure eight carcass and pork quality traits. Whole-transcriptome sequencing analysis was performed using longissimus dorsi samples of six low- and high-IMF samples; 34 ceRNA networks, based on 881, 394, 158 differentially expressed (DE) lncRNAs, miRNAs, and mRNAs, were constructed. Following weighted gene co-expression network analysis between the low and high IMF, only one ceRNA, *lncRNA4789/miR-381-3p/FABP3*, that showed similar DE trend in longissimus dorsi tissue was retained. Dual-luciferase reporter assays further indicated that *FABP3* was a direct, functional target of *miR-381-3p*, where *miR-381-3p* overexpression inhibited the mRNA and protein expression of *FABP3*. In addition, overexpressed *lncRNA4789* attenuated the effect of *miR-381-3p* on *FABP3* by sponging *miR-381-3p*. Cell function verification experiment demonstrated that *miR-381-3p* suppressed IMF deposition by inhibiting preadipocyte cell differentiation and lipid droplet deposition via the suppression of *FABP3* expression in the peroxisome proliferator-activated receptor signalling pathway, whereas *lncRNA4789* rescued *FABP3* expression by sponging *miR-381-3p*. Our study may aid in identifying novel molecular markers for its optimization in IMF which is of importance in breeding for improving pork quality.

## 1. Introduction

Pork meat accounts for 40% of the world’s meat consumption and is one of the main sources of protein, fat, and iron for humans (FAO, Rome, Italy, http://www.fao.org/faostat/zh/#data/QL) (accessed on 13 June 2021). Its quality influences consumer preferences; a high level of marbling or intramuscular fat (IMF) content is positively correlated with meat juiciness, flavour intensity, and tenderness [1,2]. Therefore, IMF content is considered a crucial indicator of porcine meat quality [3]. Over the last few decades, the parameters for pig selection have been on growth traits and lean meat rate; Western pigs were widely used for commercial production because of their high growth rate and lean meat percentage. However, long-term selection and negative correlation between lean meat rate and IMF led to the deterioration of meat quality in Western pigs, whereas Chinese indigenous pig breeds had higher IMF, increased tenderness, and better meat quality than Western pigs [4,5,6]. Presently, improvements in IMF by normal breeding programmes are challenging to gauge since it is measured post-slaughter. Thus, there is a need to invent other measures to increase IMF content and improve pork quality; this has remained an important topic of interest in recent years.

To date, the deposition of IMF is achieved through the proliferation and differentiation of intramuscular preadipocytes [7]; the proliferation of preadipocytes is regulated by cell cycle regulators including cyclin-dependent kinases (CDKs) [8], CDK inhibitors [9], and other transcription factors [10]. The differentiation of preadipocytes also involves several regulatory factors such as the peroxisome proliferator-activated receptor γ (*PPARγ*) [11], CCAAT/enhancer binding protein (*C/EBPα*) family [12], fatty acid binding protein 3 (*FABP3*) [13], and lipoprotein lipase (LPL) [14]. The above candidate genes related to pig fat content have also been studied in terms of their potential use as molecular markers for the selection of pork quality traits [15].

In addition to the above key genes that influence adipogenesis, emerging evidence suggests that microRNAs (miRNA) are also associated with intramuscular preadipocyte proliferation and differentiation in various livestock such as bovine [16], sheep [17] and chickens [18]. In porcine adipogenesis, *miRNA-196a/b* [19], *miR-32-5p* [20], *miR-15a/b* [21], *miR-196a* [22], and *miR-146a-5p* [23] promoted porcine preadipocyte differentiation, whereas *miR-34a* [24,25], *miR-451* [26], *miR-125a-5p* [27], *miR-425a-5p* [28], *miR-375* [29], and *miR-429* [30] impaired porcine preadipocyte differentiation. Furthermore, recent research indicates that long non-coding RNA (lncRNA) also plays a role in pig IMF which regulate preadipocyte differentiation or adipogenesis [10,31,32] by competing with the endogenous RNA (ceRNA) mechanism [33]. High-throughput transcriptome sequencing technology is a powerful tool for exploring ceRNA regulation of fat deposition at the molecular level in pig breeds; ncRNAs were considered as important regulatory factors for meat quality and pig breeding.

Many ncRNAs have been identified in porcine fat deposition; some of these ncRNAs remain unidentified in Chinese indigenous pig breeds, especially in Anhui, China, which is a repository of indigenous pig germplasm. The Anqin Six White pig (AQSW) is a typical Chinese local variety with high fat deposition and good meat quality, widely distributed in Anqin, Anhui province, China. In recent years, AQSW sows were crossed with Berkshire boars (Ber × AQSW) to produce high-quality meat which resulted in large intra-population variations of fat deposition and meat quality [34,35]. Therefore, Ber × AQSW crossbred population is an excellent and suitable porcine model to determine ceRNA regulation mechanism which could help identify potential target genes and key regulatory factors (miRNA and lncRNA) that affect IMF deposition in swine.

The objective of the current study was to screen for potential target genes and key regulatory factors (miRNA and lncRNA) that may have significant effects on IMF deposition in pigs. The differential expression of genes between high and low IMF in a Ber × AQSW crossbreed population could be deciphered via whole-transcriptome sequencing. Furthermore, the ceRNA-based-regulatory mechanism underlying IMF deposition was verified at the cellular level. The results of our study in a Ber × AQSW crossbred population should enhance future exploration of the hitherto unrecognised role played by the ceRNA network in IMF deposition among indigenous pig breeds. In addition, this study also allowed us to seek novel molecular markers for the optimization of IMF which is of important breeding and market economic value for improving pork quality.

## 2. Materials and Methods

### 2.1. Ethics Approval

The animal study protocol was approved by the Animal Research Committee of Anhui Academy of Agriculture Sciences (protocol code AAAS2021034 and 2021/9/2) for studies involving animals.

### 2.2. Samples and Phenotype Measure

In this study, 52 individuals were selected from the population of Berkshire *×* Anqinliubai (Ber × AQSW). These pigs were raised in the same environment with the same feeding conditions. Pigs were weighed and slaughtered (CO_2_ stunning, exsanguination, vapor scalding, and dehairing) in a commercial slaughterhouse. The average age was 180 ± 7 days and average live weight was 102.06 ± 6.622 kg. Tissue samples were collected from the same position (10th to 11th ribs) of the longissimus dorsi muscle of pigs while heart, liver, spleen, lungs, kidney, fat, and backfat were also collected at the same time. All tissues were immediately frozen in liquid nitrogen and stored in a refrigerator at −80 °C for further analysis.

Carcass traits: Eight carcass traits were determined which contained live and carcass weight (kg), average backfat (BFT, shoulders, thoraco-lumbar junction and lumbar-caudal junction) (mm) and 10th/11th ribs backfat (mm), loin eye area (cm^2^), carcass lengths (cm), carcass straight (cm) and carcass diagonal length (cm), based on the technical regulation for test of carcass traits, published by the Ministry of Agriculture, China (http://www.moa.gov.cn/nybgb/2004/dshiq/201806/t20180624_6153046.htm, accessed on 20 October 2004).

Pork quality traits: Eight pork quality traits were determined which contained IMF (%; Soxhlet extractor method), 24 h Drip loss (%), Marbling Score, Total Protein (%; FoodScan), 45 min and 24 h meat colour (lightness, L*; Minolta CR-300 colorimeter), 45 min and PH value (pH-Star), based on the technical regulation for determination of pork quality traits, published by the Ministry of Agriculture, China (http://www.moa.gov.cn/nybgb/2004/dshiq/201806/t20180624_6153046.htm, accessed on 20 October 2004).

**Figure 1:**
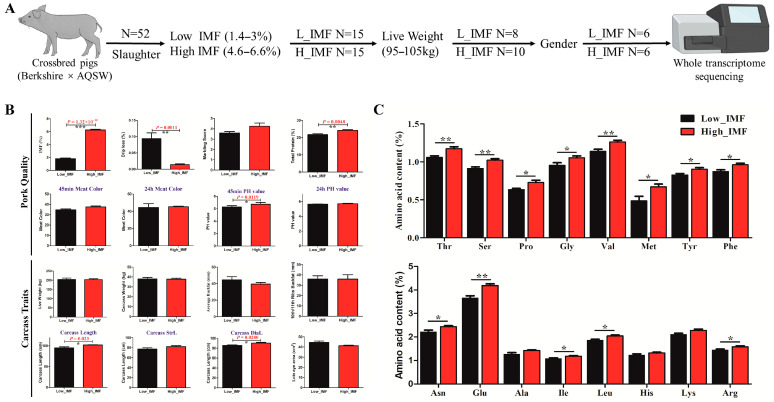
High/low IMF sample selection and multiple phenotype (pork quality and carcass traits) measure in crossbred pigs (Berkshire × AQSW). (**A**) Schematic diagram of six high/low IMF samples selected from 52 crossbred pigs (Berkshire × AQSW). (**B**) The summary of phenotype measure in pork quality and carcass traits between six high- and low-IMF samples (Berkshire × AQSW). (**C**) The phenotype measure summary of 16 essential amino acids between six high- and low-IMF samples (Berkshire × AQSW); * *p* < 0.05; ** *p* < 0.01.

Whole-transcriptome sequencing samples selection: The whole-transcriptome sequencing samples were selected based on the IMF content from all 52 Ber × AQSW samples. According to the selection criteria (Figure 1A), 15 high- and low-IMF content individuals were selected preliminarily. The IMF content ranged between 4.6–6.6% in high IMF groups whereas low IMF content (1.4–3%) in low IMF group. Furthermore, similarly live weight (95–105 kg) and same numbers of boar/sow were considered, of which six (castration boar = 3, sow = 3) high- (content = 5.8–6.6%) and low- (content = 1.4–2.2%) IMF content samples were finally selected for whole-transcriptome sequencing.

### 2.3. RNAs (mRNA and ncRNA) Sequencing and Analysis 

Total RNA was extracted using Trizol reagent (Invitrogen, Carlsbad, CA, USA), according to the manufacturer’s instructions. Quantity and purity were analysed using a Bioanalyzer 2100 and an RNA 6000 Nano Labchip Kit (Agilent, Palo Alto, CA, USA). Only RNA samples with suitable RNA electrophoresis results (28 S/18 S ≥ 1.0) and RNA integrity number (RIN) ≥ 7.5 could be analysed further. The longissimus dorsi tissues of high- (*n* = 6) and low- (*n* = 6) IMF content were selected for mRNA, lncRNA and miRNA sequencing. The mRNA and lncRNA were sequenced on Illumina HiSeq 4000 and miRNA on HiSeq 2000 platforms, which generated 150-bp paired-end and 50-bp single-end reads, respectively. Differentially expressed (DE) ncRNAs and mRNAs were identified using HISAT2 (DaehwanKim Lab, Maryland, USA) [36] and DESeq2 (Simon Anders Lab, Heidelberg, Germany) [37] with an adjusted *p* value (false discovery rate, FDR) < 0.05 and a log2|FoldChange| > 1.

mRNA sequence analysis: An Illumina high-throughput platform was used for mRNA sequencing and raw data were obtained using FastQC v0.11.9 (Babraham Institute Bioinformatics Group, Cambridge, England) [38]. Quality control of the raw reads was conducted using NGSQCtoolkit_v2.3.3 (Functional Genomics and Bioinformatics Lab, New Delhi, India) [39]. The Sus scrofa 11.1 reference genome (http://ftp.ensembl.org/pub/release-104/fasta/sus_scrofa/dna/Sus_scrofa.Sscrofa11.1.dna.toplevel.fa.gz, accessed on 11 October 2021) and gene sets file (http://ftp.ensembl.org/pub/release-104/gtf/sus_scrofa/Sus_scrofa.Sscrofa11.1.104.gtf.gz, accessed on 11 October 2021) were downloaded from Ensemble. HISAT2 (v2.0.4) and StringTie (v1.3.1) (Mihaela Pertea Lab, Baltimore, USA) [40] were performed to obtain clean reads aligned to the reference genome and mRNA reads were assembled for each sample. DESeq2 [37] package was used for differentially gene expression analysis.

lncRNA-seq analysis: Quality control and mapping methods of lncRNA-seq data were similar to those used for mRNA-seq data. Candidate lncRNAs were selected using the following conditions: (i) Transcript length ≥200 and exon number ≥2, and Fragments Per Kilobase per Million reads (FPKM) > 0.5; (ii) Minimal read coverage ≥3 in at least one sample; (iii) Filter known non-lncRNA annotations; and (iv) Classify selected candidate lncRNAs, using the following four tools to predict coding potential: CPC2 [41], CNCI [42], PhyloCSF (v20121028) [43], and Pfam Scan databases [44]. DESeq2 package was used to identify differentially expressed lncRNA between different groups at padj <0.05. The lncRNA function depends on protein coding genes via cis- and trans-acting elements. Cis-acting (in which lncRNAs act on adjacent genes within a 100 kb distance) and trans-acting elements (in which the Pearson correlation of mutual expression levels is ≥0.95 or ≤−0.95) are widely adopted to forecast lncRNA gene interactive pairs. Bedtools [45] was used to identify neighbouring genes approximately 100 kb upstream and downstream of differentially up-regulated and down-regulated lncRNAs, respectively.

microRNA-seq analysis: Quality control methods used for miRNA-seq data were similar to those used for mRNA-seq data, where an NGSQCtoolkit_v2.3.3 was employed. BWA software [46] was used for mapping and pig reference miRNA. Novel miRNA was predicted via miRDeep2 [47]. The DESeq2 package was performed to identify miRNAs that were differentially expressed between different groups and a screening threshold for identifying differentially expressed miRNA was established at padj < 0.05. The target genes of miRNAs were predicted using Target Scan (http://www.targetscan.org/mamm_31/, accessed on 2 December 2021), RNA hybrid (https://bibiserv.cebitec.uni-bielefeld.de/rnahybrid/, accessed on 2 December 2021) and miRWalk v3.0 database (http://mirwalk.umm.uni-heidelberg.de/, accessed on 2 December 2021). Google charts (https://developers.google.com/chart/, accessed on 2 December 2021) were used to illustrate the targeting relationship between miRNA and mRNA. GO and Pathway enrichment analysis was performed using g:Profiler (https://biit.cs.ut.ee/gprofiler/gost, accessed on 2 December 2021) and ToppGene (https://toppgene.cchmc.org/enrichment.jsp, accessed on 2 December 2021), in which terms with an adjusted *p* value (false discovery rate, FDR) greater than 0.05 were filtered.

### 2.4. Integrated ceRNA Regulatory Network

To investigate the role and interactions between ncRNAs and mRNAs during IMF deposition, ceRNA regulatory networks were constructed. The targeted relationships between lncRNA and miRNA were predicted via miRanda. Next, regulatory networks of lncRNA–miRNA–mRNA pairs were constructed based on co-location and co-expression [33]. Two expression trend models, namely lncRNA down-regulated |miRNA up-regulated |mRNA down-regulated (lncRNA−|miRNA+|mRNA−) and lncRNA up-regulated |miRNA down-regulated |mRNA up-regulated (lncRNA+|miRNA−|mRNA+) were used to establish ceRNA regulatory networks.

### 2.5. Weighted Gene Co-Expression Network Analysis (WGCNA)

The mRNA sequencing of 12 longissimus dorsi tissues with high and low IMF were integrated into the gene expression matrix for WGCNA. Then the mRNA-seq data, which contained the expression data of 13,215 genes (Sum counts > 10) for WGCNA, were used to construct the gene expression matrix. Variance stabilizing transformation (VST) (Anders and Huber, 2010) function in the DESeq2 package was used to normalize and transform the two gene expression matrices, respectively. The construction of two gene co-expression networks was based on the WGCNA package [48]. Gene co-expression networks must conform to scale-free characteristics and obey power law distribution. Following sensitivity analysis of scale-free topology, the soft threshold power parameters of the networks were set at 21 [49]. The interaction network of *FABP3* was constructed by STRING [50], while different species of *miR-381-3p* sequence alignment analysis were performed.

### 2.6. Primary Preadipocytes Isolation, Culture

The experiments refer to in vitro intramuscular adipocytes from Berk × AQLW pigs. Intramuscular preadipocytes (preADs) were isolated from the longissimus dorsi muscle of 3-day-old piglets. The longissimus dorsi muscle was dissected, washed with DPBS (HyClone), supplemented with 5% penicillin–streptomycin (P/S), minced, and digested for 60 min at 37 °C in Dulbecco’s Hanks Balanced Salt Solution (D-Hanks) (Solarbio) containing 0.2% type I collagenase digestion solution (Gibco, Carlsbad, CA, USA). Then, samples were sequentially filtered through 70 and 200 mesh filters to separate the cells, and centrifuged at 1500 r/min for 10 min at room temperature. The upper mature adipocytes were carefully aspirated, and DMEM was added before washing, resuspending, and centrifuging again. After, cells were seeded in 25 cm^2^ culture flask containing DMEM/F12 medium (SH30023.01, GE HyClone) with 10% fetal bovine serum (Gibco, Australia) and maintained at 37 °C in a humidified environment containing 5% CO_2_. Around 10 days later, a large number of fibrous, dedifferentiated pre-adipocytes were observed and the culture medium was discarded and converted to normal culture.

### 2.7. Primary Preadipocytes Differentiation

After reaching 80% confluence, cells were digested with 0.05% trypsin and seeded in 12-well plates (2 × 10^5^ cells/well) for further differentiation research. For preadipocyte differentiation, cells were treated with induction medium (Cyagen Biosciences, Santa Clara, CA, USA). The induction medium was composed of medium A (fetal bovine serum (FBS), penicillin/streptomycin, insulin, glutamine, 3-isobutyl 1- methylxanthine, rosiglitazone, and dexamethasone) and medium B (FBS, penicillin/streptomycin, and insulin), which were alternately used every two days until day 8 for cell culture.

### 2.8. Oil Red O Staining and Immunofluorescence Staining

PreAD cells differentiation for 0, 2, 4, 6, and 8 days were washed three times with PBS and fixed in 4% paraformaldehyde solution and incubated with 0.5% Oil Red O for 1 h. After cell samples were further washed three times with PBS, images were visualized via phase-contrast microscope (IS-Elements software, Nikon ECLIPSE, Tokyo, Japan) by measuring the absorbance at 510 nm. Cells were seeded, cultured and differentiated in glass-bottom confocal plates (D35-20-0-N, Cellvis). On the day of the experiment, 200 nM of BODIPY™ 493/503 (D3922, Thermo Fisher Scientific, Waltham, MA, USA) used to stain lipid droplets was added to the cell culture medium for 30 min. Imaging was performed on Lipofectamine 3000 (Invitrogen, Carlsbad, CA, USA).

### 2.9. Transfections

To evaluate the effect of *miR-381-3p* on the differentiation of porcine intramuscular preadipocytes, cells were seeded in 12-well or 6-well plates and transfected, respectively, with negative control of *miR-381-3p* mimics (mimics-NC, *n* = 3), *miR-381-3p* mimics (*n* = 3), 100 pmol of negative control of *miR-381-3p* inhibitor (inhibitor-NC, *n* = 3) or *miR-381-3p* inhibitor (*n* = 3), and 20 pmol of negative control *FABP3* siRNA (*FABP3*-NC, *n* = 3) or *FABP3* siRNA (*n* = 3), using Lipofectamine 3000 (Invitrogen, Carlsbad, CA, USA) according to the manufacturer’s instructions. The siRNA, mimics and inhibitor were designed and synthesized by RIBOBIO (Guangzhou, China). Total RNA and protein were isolated from the transfected cells 48/72 h post-transfection for further analysis.

### 2.10. Luciferase Reporter Assay

The *miR-381-3p* target gene and miRNA binding sites were identified using the Target Scan software (David Team, Cambridge, USA). The mature sequence of *miR-381-3p* was obtained from the miRBase database (https://www.mirbase.org/, accessed on 2 December 2021). HEK293T cells (National Biomedical Cell Collection, Beijing, China) were cultured in 24-well plates, and plasmids were transfected when the cell reached 70% or 80% confluence. Luciferase activities were measured 48 h after transfection on a Fluoroskan Ascent FL instrument (Thermo Fisher Scientific, Waltham, MA, USA) by using the Dual-Luciferase Reporter Assay System (Promega, Madison, WI, USA).

### 2.11. Western Blotting

Western blotting (WB) was used to detect protein quantity. In brief, the sample was lysed in RIPA lysis solution to extract total protein and denatured. Next, the sample proteins were separated via SDS-PAGE gel electrophoresis under conditions involving a constant voltage of 100 V. In this study, the proteins were transferred from SDS-PAGE gel to PVDF membranes using a semi-dry transfer method. The membranes were then blocked for 4 h, and incubated with primary antibodies Anti-*CEBPα* (Abcam, 1:1000) Anti-*PPARγ* (Abcam, 1:1000) and *FABP3* (Cell Signaling, 1:1500) overnight at 4 °C. Next, the membranes were washed thrice with TBST (Tris-buffered saline) and incubated with secondary antibody, rabbit anti-goat IgG antibody (Novogene, 1:5000) at room temperature for 1.5 h. Finally, a BeyoECL plus kit (Beyotime) was used to detect the protein signal. β-tubulin (Cell Signaling, 1:2000) was used as a reference protein and blots were analysed using IPWIN software.

### 2.12. Quantitative RT-PCR Analysis

Total RNA was isolated from dorsal pig skin with TriZol (Invitrogen, SanDiego, CA, USA) following the manufacturer’s instructions. Next, cDNA was reverse transcribed using a PrimeScriptTM RT reagent kit with gDNA Eraser (Takara, Kyoto, Japan). RT-PCR was performed with LightCycler 480 SYBR Green I Master (Roche, Mannheim, Germany) mix on a LightCycler 480 real-time PCR system. GAPDH was used as a normalized control and relative gene expression was calculated based on the 2^−ΔΔCt^ formula. Measurements were recorded in triplicate. The following PCR conditions used: 95 °C “hot start” for 10 min; 35 cycles of 95 °C for 10 s, 60 °C for 10 s, and 72 °C for 10 s; and 72 °C for 5 min. Primer sequences are provided (Supplementary File Table S1). Differences between the gene expression of high and low IMF were measured via a *t* test.

### 2.13. Statistic Analysis

Data analysis was performed in GraphPad v.8 (GraphPad Software Inc., San Diego, CA, USA). Data are presented as mean ± SD. Each experiment was performed in triplicate. Student’s t-test was used for pairwise comparisons. Statistical significance was considered at values of * *p* < 0.05; ** *p* < 0.01, *** *p* < 0.001.

## 3. Results

### 3.1. Phenotype Measure of Pork Quality and Carcass Traits in Ber × AQSW Crossbred Population

To select individual pigs with contrasting low and high IMF, 52 Ber × AQSW crossbred pigs were sacrificed at 180 days with the measurement of eight pork quality and carcass traits in all pigs. The summary of phenotypes for pork quality and carcass traits are represented in Table 1. While six high- and low-IMF samples were collected based on their contrasting IMF content, they were selected to be of similar live weight, and with the same numbers of boar/sow (castration boar = 3, sow = 3) (Figure 1A). As shown in Figure 1B, a significant difference in IMF percentage (*p* = 1.32 × 10^10^), drip loss (*p* = 0.0011), total protein (*p* = 0.0048) and 45 min pH value (*p* = 0.0119) among carcass traits was observed; a significant difference was also observed in carcass length (*p* = 0.023) and carcass diagonal length (*p* = 0.028) under pork quality traits between IMF high/low samples. Furthermore, 16 essential amino acids were detected between high/low IMF (Supplementary File Table S2); high-IMF samples showed an increase in amino acid content except Ala, His, and Lys (Figure 1C). These results demonstrated that our collection of high- and low-IMF samples was accurate and reliable to be used for whole-transcriptome sequencing analysis.

### 3.2. Specific Differential Expression of mRNA and ncRNAs between High and Low IMF

**Figure 2:**
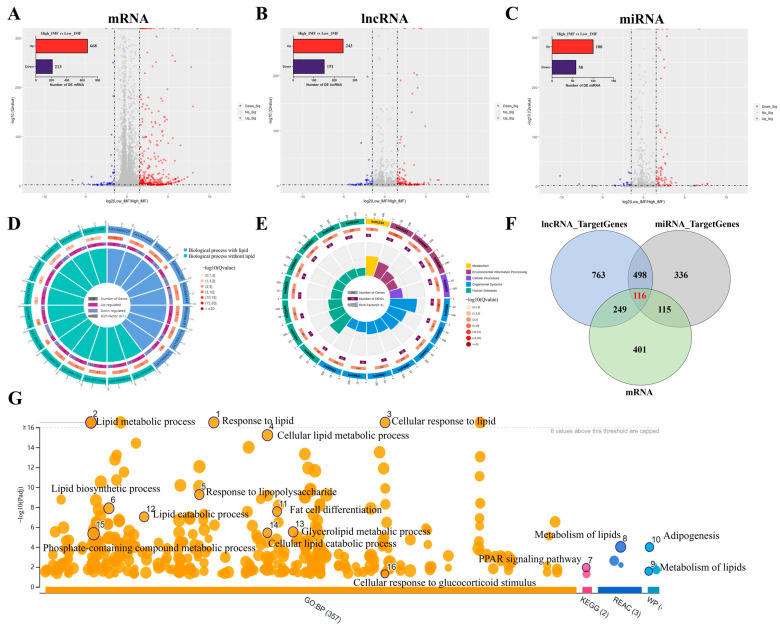
Differential expression of mRNAs, miRNAs, and lncRNAs between high and low IMF in crossbred pigs (Berkshire × AQSW). Volcano plots for the mRNAs (**A**), miRNAs (**B**) and lncRNAs (**C**) with up-regulated and down-regulated expressions between high and low IMF in crossbred pigs (Berkshire × AQSW). (**D**) GO enrichment analysis for DE mRNAs. (**E**) KEGG enrichment analysis for DE mRNAs. (**F**) Venn diagram of candidate target genes of DE mRNA, DE miRNA and DE lncRNA between high and low IMF. (**G**) Top 20 terms of GO and KEGG enrichment in 116 overlapped target genes.

**Figure 3:**
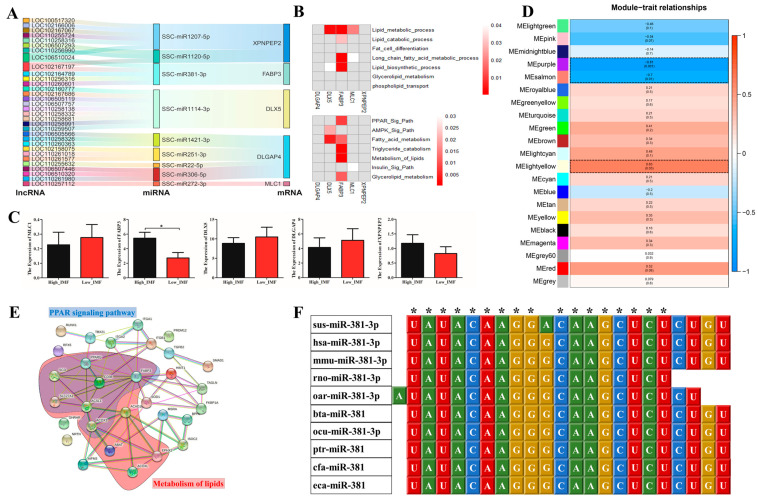
ceRNA regulatory network construction and WGCNA between high and low IMF in crossbred pigs (Berkshire × AQSW). (**A**) Sankey diagram of the ceRNAs network in longissimus dorsi tissue. Each rectangle represents a gene, and the degree of connection of each gene is expressed based on the size of the rectangle. (**B**) GO and KEGG enrichment analysis of the five DE target genes in ceRNAs; (**C**) the mRNA expression of five DE target genes in longissimus dorsi tissue between high and low IMF. (**D**) Module–trait associations of longissimus dorsi tissue between high and low IMF. Each row corresponds to a module epigene, while each column corresponds to IMF. Each cell contains the corresponding correlation and *p*-value. The table is colour-coded by correlation according to the colour legend. The black solid box represents the significance of the modules associated with IMF. (**E**) The interaction network of *FABP3* in the MElightyellow module of the IMF. The hub genes of *PPAR* signalling pathway and lipids metabolism in the modules are denoted by blue and red shadow. (**F**) Sequence alignment analysis of *miRNA-381-3p*. sus, Sus scrofa; hsa, Homo sapiens; mmu, Mus musculus; rno, Rattus norvegicus; oar, Ovis aries; bta, Bos taurus; ocu, Oryctolagus cuniculus; ptr, Pantroglodytes; cfa, Canis familiaris; eca, Equus caballus; * *p* < 0.05.

A summary of the descriptive statistics of lncRNA-seq, miRNA-seq, and mRNA-seq data pertaining to high/low groups indicated the relatively high-quality of transcriptome data in this study (Supplementary File Tables S3 and S4). To identify intra-group consistency and inter-group variability, principal component analysis was performed; the results showed that the samples between high and low IMF can be clearly divided into two groups (Supplementary File Figure S1). Furthermore, the number of differentially expressed (DE) mRNAs and ncRNAs are shown in Table 2 while the DE RNAs (mRNAs, miRNAs, and lncRNAs) are illustrated in detail (Supplementary File Table S5). Sample-to-sample correlation showed that the heatmap between high/low IMF was high (Supplementary File Figure S2). Overlaps in expression profiles among DE mRNAs, DE miRNAs and DE lncRNAs are illustrated (Figure 2A–C). In addition, 881 DE mRNAs (Figure 2A), 158 DE miRNAs (Figure 2B), and 394 DE lncRNAs (Figure 2C) were identified between high and low IMF. The qRT-PCR of expression levels in randomly selected DE mRNA and ncRNA transcripts showed that their expression levels were highly consistent (Supplementary File Figure S3), indicating the reliability of our RNA-seq data. In total, eight GO terms (Figure 3D and Supplementary File Table S6) of the 881 DE mRNAs were mainly enriched in lipid metabolic and catabolic processes, fat cell differentiation and long-chain fatty acid metabolic processes, while seven lipid-related signalling pathways, including the *PPAR*, *AMPK*, *FoxO* and *insulin signalling pathway*, were identified (Figure 2E and Supplementary File Table S7). A total of 1626 *cis* (487) or *trans* (1139) target genes were predicted for the 394 DE lncRNAs in the longissimus dorsi, of which 365 target genes overlapped with the DE mRNAs (Supplementary File Figure S4A left panel). Similarly, 1065 overlapped target genes were predicted for the 158 DE miRNAs by miRanda (4064 target genes) and RNAhybrid (3182 target genes), of which 231 target genes overlapped with the DE mRNAs and miRNAs (Supplementary File Figure S4B left panel). Furthermore, GO terms such as lipid metabolic process, fat cell differentiation, phospholipid transport and pathways including sphingolipid, glucagon, insulin/*AMPK* signalling pathway among others, were enriched in these overlapped target genes (Supplementary File Figure S4A,B right panel). The 116 genes that overlapped between high and low IMF in the longissimus dorsi were identified based on DE mRNAs and target genes of DE miRNAs and lncRNAs (Figure 2F and Supplementary File Table S8) in which these genes had an interaction communication in different biological processes (Supplementary File Figure S5). These were enriched in lipid-related biological processes, including lipid metabolic and biosynthetic process, adipogenesis, fat cell differentiation, and glycerolipid metabolic processes; similarly, enriched in the classical lipid pathways was the *PPAR* signalling pathway (Figure 2G and Supplementary File Table S9) in which the key genes such as *NR4A3*, *CPT1A*, *LIPE*, *PCK1*, *SPP1*, *FABP3* and *ANGPTL4* among others, were identified.

### 3.3. Construction and Weighted Gene Co-Expression Network Analysis (WGCNA) of ceRNA Regulatory Networks

Based on the expression trend models (lncRNA−|miRNA+|mRNA− or lncRNA+|miRNA−|mRNA+) pertaining to 116 overlapping target genes as well as the lncRNAs and miRNAs regulating the expression of these genes, 34 ceRNA regulatory networks were constructed. These networks contained five overlapped DE genes, 32 DE lncRNAs, and nine DE miRNAs (Figure 3A and Supplementary File Table S10). Function and pathway enrichment analysis of GO (Figure 3B) showed that *FABP3* and *DLX5* were enriched in classical lipid pathways such as *PPAR*, *AMPK*, fatty acid and triglyceride metabolism signalling pathways; *FABP3* was related to lipid metabolic, catabolic, and biosynthetic processes while *DLX5* and *MLC1* were related to the GO term of lipid metabolic process. Moreover, the mRNA expression levels of five target DE genes between high and low IMF in longissimus dorsi tissues were detected and the results showed that only *FABP3* had a significantly higher expression (*p* = 0.023) in the high-IMF tissue (Figure 3C). To further screen ceRNAs for lipid-specific expression in longissimus dorsi, WGCNA was performed on high and low IMF using mRNA sequencing. The independence degree was approximately 0.85 while the average connectivity degree was higher (Supplementary File Figure S6A). In total, 21 distinct gene co-expression modules were constructed for the high- and low-IMF groups, respectively (Supplementary File Figure S6B). Modules showing *p* < 0.05 were selected, and interestingly, three modules, *MEpurple* (*p* = 0.001), *MEsalmon* (*p* = 0.01), and *MElightyellow* (*p* = 0.03), were identified between high and low IMF (Figure 3D). Among the hub genes of the three significant modules, only *FABP3* (Figure 3E) of the five DE target genes in ceRNA was found to be involved. Additionally, *FABP3* interacted with many hub genes (such as *PPARG*, *CD36*, *SCD*, *SCL27A1* and *ACSL1*) which were related to *PPAR* signalling pathway and lipids metabolism, in *MElightyellow* module (Figure 3D). Therefore, five ceRNAs containing *miR-381-3p*/*FABP3* were retained. The results of *miR-381-3p* sequence alignment in different species revealed that *miR-381-3p* is highly conserved among the species (Figure 3F), suggesting the significance of the role played by *miR-381-3p*. In summary, the results of our current study indicated that *FABP3* may play an important role in IMF deposition via the ceRNA network.

### 3.4. FABP3 Positively Correlated with the Adipogenic Differentiation Capacity of preAD Cells

**Figure 4:**
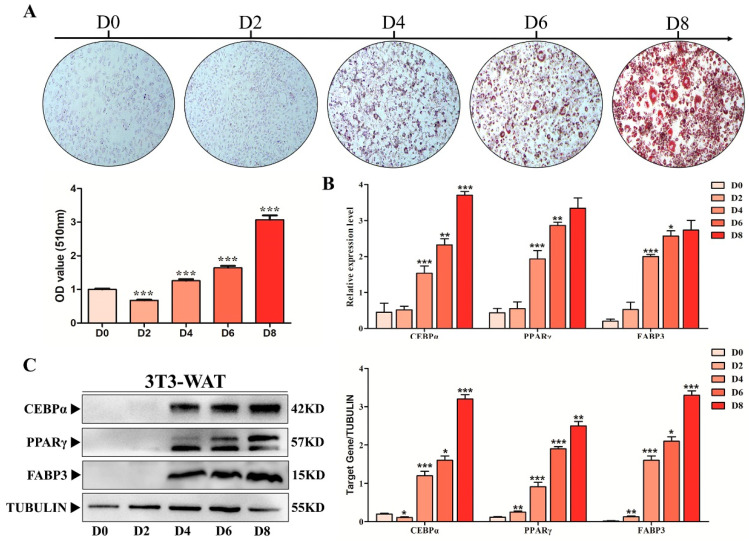
*FABP3* positively correlated with the adipogenic differentiation. (**A**) ORO staining and quantification gradually increased from 0 to 8 days (scale bar = 150 mm). (**B**) The mRNA expression levels of *PPARγ*, C/EBPα and *FABP3* during adipogenic differentiation. (**C**) The protein expression levels of *PPARγ*, C/EBPα and *FABP3* during adipogenic differentiation; * *p* < 0.05; ** *p* < 0.01, *** *p* < 0.001.

The preAD cells were cultured in adipogenic medium for up to 8 days (D8), and Oil Red O (ORO) staining was performed to detect the adipogenic differentiation of preAD cells. As shown in our results, ORO staining significantly increased (*p* < 0.001) gradually from D0 to D8 while similar results were obtained with the quantification of ORO staining (Figure 4A). Moreover, the mRNA and protein expression of key adipogenic markers, including *PPARγ* and *CEBPα* were upregulated during adipogenic differentiation within D8 (Figure 4B,C). Consistent with *PPARγ* and *CEBPα*, *FABP3* expression was also significantly increased during preAD cell differentiation from D0 to D8. All these results indicate that *FABP3* might play a key role in the preAD differentiation process similar to the adipogenic markers, *PPARγ* and *CEBPα*.

### 3.5. FABP3 Was a Target Gene of miR-381-3p while lncRNA4789 Released FABP3 by ceRNA

**Figure 5:**
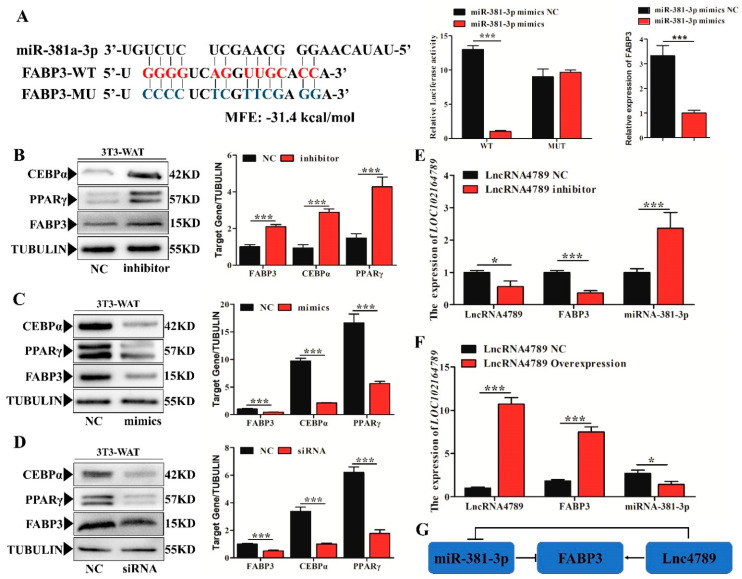
Verification of *miR-381-3p* target binding for *FABP3* 3′ UTR while *lncRNA4789* released the expression of *FABP3* by ceRNA. (**A**) Left histogram: Prediction of binding sites between *miR-381-3p* and *FABP3* in RNAhybrid. Right histogram: left panel showed luciferase assays were performed in 293-Tcells co-transfected with pMirGLO-*FABP3*–3′-UTR-WT/MUT and *ssc-miRNA-381-3p* mimics while right panel showed *FABP3* mRNA expression detected in adipocyte after treatment with *ssc-miRNA-381-3p* mimics. Red letters indicate wild type sites and blue letters indicate mutated sites in the pMir-report luciferase reporter vector; protein expression levels of *PPARγ*, *C/EBPα* and *FABP3* between NC and *miR-381-3p* inhibitor; (**B**) *miR-381-3p* mimics (**C**) and *FABP3*-siRNA; (**D**) mRNA expression of *FABP3* and *miR-381-3p* under *lncRNA4789* overexpression (**E**) and the presence of an inhibitor (**F**); (**G**) ceRNA regulation mechanism in *miRNA-381-3p*, *FABP3* and *lncRNA4789*. Error bars indicate the mean ± SD of triplicate experiments; * *p* < 0.05, *** *p* < 0.001; MFE, minimum free energy.

Among the ceRNAs, only *miR-381-3p* could regulate *FABP3* expression. To validate direct regulation of the expression levels of *FABP3* by *miR-381-3p*, a construct containing the 3′ UTR of the potential target genes, or the sequence with the mutant seed region, was co-transfected together with *miR-381-3p* mimics in human 293T cells. As predicted, *miR-381-3p* mimics significantly reduced *FABP3*-WT-3′ UTR (*p* < 0.001) luciferase activity, whereas no significant inhibition of *FABP3*-MUT-3′ UTR was detected (Figure 5A). Furthermore, the protein expression levels of *FABP3* and adipogenic markers, *PPARγ* and *CEBPα* were significantly increased (*p* < 0.001) by a *miR-381-3p* inhibitor (Figure 5B) whereas these levels were significantly decreased (*p* < 0.001) by *miR-381-3p* mimics (Figure 5C) which was consistent with the result of *FABP3* siRNA treatment (*p* < 0.001) (Figure 5D), when compared with that in the control group (NC). Moreover, the competitive mechanism involving *lncRNA4789*-*miR-381-3p*-*FABP3* is illustrated (Figure 5E), where the *lncRNA4789* inhibitor significantly upregulated (*p* < 0.001) *FABP3* expression while downregulating *miR-381-3p* expression (*p* < 0.001). Meanwhile, the *lncRNA4789* overexpression group displayed a contrasting expression trend, where *FABP3* was downregulated and *miR-381-3p* was upregulated (Figure 5F).

Our results confirmed that *FABP3* is a direct and functionally relevant target of *miR-381-3p*, whereas *lncRNA4789* could release the expression of *FABP3* by ceRNA regulation of *miR-381-3p* (Figure 5G). Thus, *FABP3* participates in IMF deposition possibly via ceRNA activity.

### 3.6. FABP3 Promotes IMF Deposition in the PPAR Signalling Pathway via ceRNA Regulation Mechanism (miR-381-3p/FABP3/lncRNA4789)

**Figure 6:**
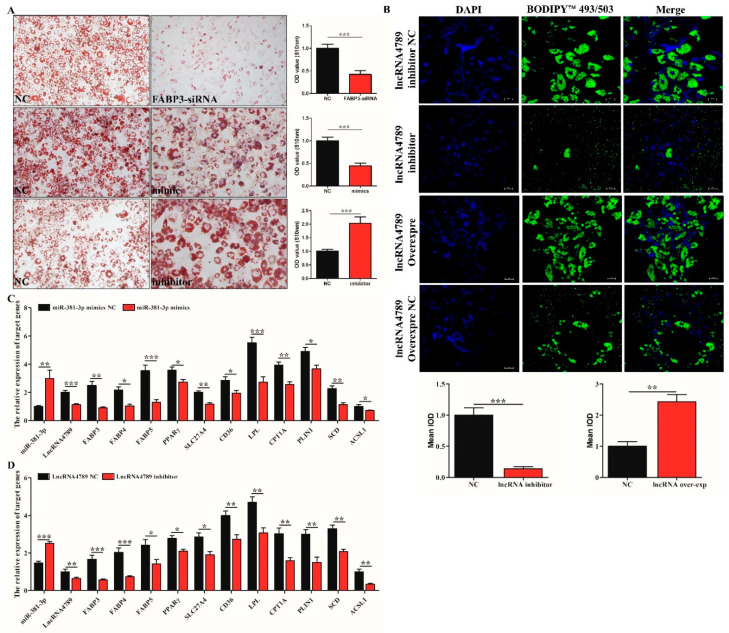
*FABP3* regulated adipogenic differentiation by *PPAR* signalling pathway. (**A**) ORO staining and quantification between NC and *FABP3*-siRNA (top), *miR-381-3p* mimics (middle) and inhibitor (bottom); (**B**) fluorescence staining of lipid droplet following transfection of *lncRNA4789* overexpression, *lncRNA4789* inhibitor, *lncRNA4789* overexpression NC and *lncRNA4789* inhibitor NC; cell nuclei were stained with DAPI (blue) while lipid droplets were stained green with BODIPY™ 493/503; (**C**) mRNA expression of related genes involved in the *PPAR* signalling pathway, following *miR-381-3p* overexpression; (**D**) mRNA expression of related genes involved in the *PPAR* signalling pathway following transfection with an *lncRNA4789* inhibitor; * *p* < 0.05, ** *p* < 0.01, *** *p* < 0.001.

**Figure 7:**
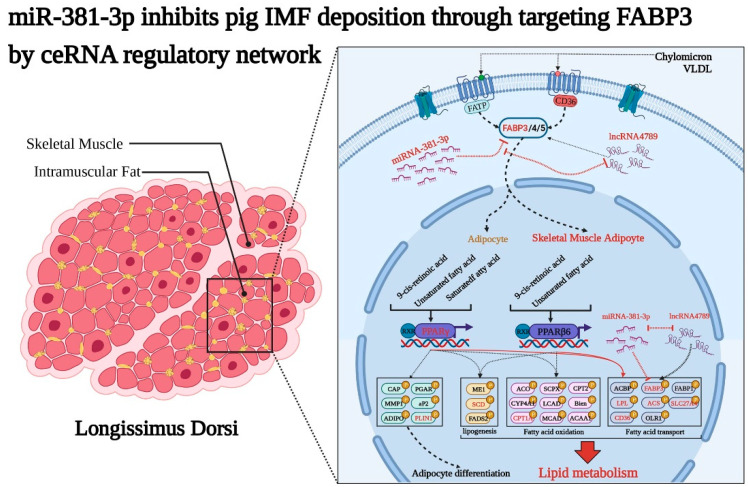
*MiR-381-3p* inhibits IMF deposition through targeting *FABP3*, while *lncRNA4789* relieves the inhibitory effects of *miR-381-3p* on *FABP3* in pig longissimus dorsi via a ceRNA regulatory network.

To further verify the function of ceRNA and *miR-381-3p*/*FABP3*/*lncRNA4789* in IMF deposition, ORO staining was performed to detect the adipogenic differentiation between NC group and *FABP3*-siRNA, *miR-381-3p* mimics, and *miR-381-3p* inhibitor group. The results showed a significantly higher (*p* < 0.001) number of preAD cells with adipogenic differentiation ability in the *miR-381-3p* inhibitor group (Figure 6A, bottom panel) while increased expression of *miR-381-3p* (Figure 6A, middle panel) and decreased expression of *FABP3* (Figure 6A, top panel) were observed as weak adipogenic differentiation. Furthermore, fluorescence staining indicated that the number of lipid droplets were significantly different between NC and *lncRNA4789* treatment groups; decreased expression of *lncRNA4789* could significantly inhibit lipid droplet formation (Figure 6B), which is similar to the results obtained in *FABP3*-siRNA and *miR-381-3p* mimics groups (Figure 6A top and bottom). To gain further insight into how *lncRNA4789* and *miR-381-3p* regulate *FABP3*, related genes, *FABP4*, *FABP5*, *PPARγ*, *SCL27A4*, *CD36*, *LPL*, *CPT1A*, *PLIN1*, *SCD* and *ACSL1* associated with the *PPAR* signalling pathway, were also detected after transfecting preAD with *miR-381a-3p* mimics and an *lncRNA4789* inhibitor. Among the *miR-381a-3p* mimics group, *FABP4* (*p* < 0.05), *FABP5* (*p* < 0.001), *PPARγ* (*p* < 0.05), *SCL27A4* (*p* < 0.01), *CD36* (*p* < 0.05), *LPL* (*p* < 0.001), *CPT1A* (*p* < 0.01), *PLIN1* (*p* < 0.05), *SCD* (*p* < 0.01) and *ACSL1* (*p* < 0.05) were downregulated (Figure 6C). Similar results were observed in the *lncRNA4789* inhibitor group (Figure 6D) while opposite expression trends were represented in *miR-382-3p* inhibitor groups (Supplementary File Figure S9A) and *lncRNA4789* overexpression groups (Supplementary File Figure S9B). Taken together, our results not only indicated that *miR-381a-3p* inhibits lipid droplet formation and deposition by suppressing the expression of the target gene, *FABP3* while *lncRNA4789* removed such an inhibition in ceRNA regulation mechanism, but also demonstrated that ceRNA regulation mechanisms were performed mainly through the *PPAR* signalling pathway (Figure 7).

## 4. Discussion

Presently, with improvements in consumer living standards, the demand for pork has changed from quantity to quality with IMF content representing an important determinant of meat quality [1,2]. Previous studies have indicated that [51,52,53] compared with Western commercial pigs (WECPs), Chinese indigenous pigs (CHIPs) displayed better pork quality traits in terms of IMF percentage, 24 h drip loss, meat colour and pH value while WECPs were better in terms of lean meat percentage (BFT and loin eye area) (Table 3). Anqing Sixwhite pig (AQSW) is an excellent breed raised in China demonstrating resistance to coarse fodder and disease, superior meat quality and high prolificacy [34,35]. As a result, CHIPs have better pork quality, where an increase in the accumulation of IMF can promote the formation of meat marble patterns and improve the taste, flavour, colour, and other characteristics of meat [54,55]. However, to overcome the shortcomings of high fat content and slow growth rate in CHIPs, WECPs were always considered as the male parent of choice while retaining the meat quality traits. Compared with WECPs and CHIPs, crossbred pigs could increase loin eye area and reduce BFT while maintaining higher IMF percentage and meat colour [53,56,57]. Thus, in our study, Berk × AQSW crossbred pigs were assessed for pork quality and carcass traits; we found the percentage of IMF and 24 h drip loss to be between Jixing black pig [53] and Duroc [58] or Yorkshire [59], while the traits were consistent with those of the other crossbred pigs such as, Meishan × Duroc [60] and Duroc × Duroc × Berkshire × Jixing black pig [53]. Amino acids are fundamental units of proteins that play a vital role in meat quality by providing nutritive value and flavour characteristics to the meat [61]. Among the 16 types of amino acids detected in this study, it was revealed that except for Ala, His and Lys, the amino acid contents of others were significantly affected by IMF; this indicated that the amino acid composition was influenced by IMF proportion (Figure 1C). Moreover, the main amino acids that act as important flavour precursors [62,63], Glu (*p* < 0.01) and Asn (*p* < 0.05) were significantly higher in the high-IMF group in our study (Figure 1C); Glu and Asn synergise with inosinic acids leading to enhancements in taste and buffering of undesirable acidic and alkaline flavours [62]. Similar reports are found in other CHIPs which were comparable to Berkshire (IMF = 3.01%); the contents of 16 kinds of amino acids in Jiaxing Black Pigs (IMF = 4.8%) except Ser and Asp were higher [53] while Qingyu pigs (IMF = 2.48% ± 0.3) had greater amino acid concentration than that of Yorkshire (IMF = 1.43% ± 0.55) [64]. Thus, a high percentage of IMF could improve the meat quality traits which not only promote the formation of meat marble patterns and meat colour, but also maintain the taste, flavour, colour, and other characteristics of meat by increases in essential amino acid concentration.

In this study, whole-transcriptome sequencing (mRNA, lncRNA and miRNA) of low- and high-IMF from the longissimus dorsi of Berk × AQSW crossbred pigs identified *FABP3* and an associated regulatory mechanism involving ceRNAs. Several studies revealed the diverse roles of *FABP3* related to cell signalling, growth inhibition, preadipocyte differentiation, fatty acid transport (during intermediate stages of adipogenesis), and fat deposition which had been reported in different species such as mouse [68], chicken [69], goat [70], and cow [71]. Additionally, in pigs, *FABP3* has been proposed as the candidate gene responsible for IMF levels, which is further associated with meat quality accounting for variations in BFT and IMF contents [72]. In WECPs, the expression levels of *FABP3* continuously increased from day 0 to 150 in longissimus dorsi tissues in Yorkshire pigs [73] whereas *FABP3* was also at a higher expression (*p* < 0.05) level in high-IMF Duroc pigs [74]. In our study, a greater *FABP3* expression was found in the longissimus dorsi tissues of the high-IMF group than that in the low-IMF (Figure 3C) group. This not only confirmed the key role of *FABP3* in IMF deposition, but also showed consistency in the results of previous studies in CHIPs such as Laiwu [72] and Banna mini pig [75]. Furthermore, this study also demonstrated that high *FABP3* expression promotes the adipogenic differentiation capacity of preAD cells, while mRNA and protein expression levels continuously increased with the deposition of lipid droplets (Figure 4B,C). Previous studies had also reported that *FABP3* exerts its effect on 3T3-L1 preAD [75] and skeletal muscle cells [68] differentiated via the *PPAR* signalling pathway. Two major differentiation markers [76], *PPARγ* and *CEBPα* were also detected which increased significantly with the consistent expression of *FABP3* (Figure 4B,C). Furthermore, in pigs, the expression of *FABP3* enhances adipogenesis in preadipocytes primarily by upregulating lipogenic *PPARγ*, 422/aP2 and GPDH genes [75], while *PPARδ* activation enhances lipid accumulation via *FABP3* and *FABP5* [77]. Thus, our study indicated that *FABP3* could directly promote IMF deposition in Berk × AQSW crossbred pigs by enhancing the adipogenic differentiation capacity of preAD in longissimus dorsi tissue.

An increasing amount of evidence indicates that ncRNA participates in the regulation of IMF deposition via the ceRNA network, which suggests the existence of mutual regulation patterns among miRNAs, lncRNAs, and mRNAs [78,79]. According to the ceRNA theory, miRNA suppresses gene expression by recognising specific target mRNAs, while lncRNAs, which act as natural miRNA sponges, inhibit miRNA function, and modulate the expression of target mRNAs by interacting with miRNA response elements [80]. In our study, significantly high expression levels of *miR-381-3p* suppressed the expression of *FABP3* and *lncRNA4789* in Berk × AQSW crossbred pigs longissimus dorsi tissue (Figure 5G). Meanwhile, overexpression of lncRNA4789 elevated the expression of *FABP3* and indicated a reduction in the expression of *miR-381-3p*, which suggested that *lncRNA627.1* may disrupt the binding between miRNA and mRNA; this could be determined using competitive binding displacement assays in future (Figure 5F). Another similar regulatory mechanism based on ceRNAs is that of lncRNA IMFlnc1, which directly promotes porcine intramuscular adipogenesis by sponging *miR-199a-5p* to upregulate CAV1 in Huainan pigs (CHEPs) [32]. In chickens, lncRNA IMFNCR promotes intramuscular adipocyte differentiation by sponging *miR-128-3p* and *miR-27b-3p* [81]. Generally, ncRNA regulates the expression of targeted mRNAs [80]; for example, Cheng et al. (2021) identified DE lncRNAs, miRNAs, circRNAs, and mRNAs and constructed multiple ceRNAs which play an important role in the complex molecular processes of IMF deposition between large white × Min crossbred pigs [82]. Previous studies have reported that *miR-381-3p* plays a positive role in adipogenic differentiation of adipose-derived stem cells [83], obesity-related diseases (type 2 diabetes) [84], and sheep muscle growth and development [85] by regulating target genes. The results of the current study further revealed that *miR-381-3p* plays a role in IMF deposition by regulating *FABP3*. *MiR-381-3p* downregulated *FABP3* expression, thereby inhibiting preAD differentiation and lipid droplet deposition. In contrast, *lncRNA4789* upregulated *FABP3* expression by suppressing *miR-381-3p* expression (Figure 6A). It is worth noting that the current data supported a model where *lncRNA4789* acts upstream of *miR-381-3p* expression (Figure 5E,F), but there is no direct evidence indicating that *lncRNA4789* could release the *FABP3* expression by competitively binding to *miR-381-3p* based on a ceRNA regulatory mechanism. Thus, the relationship between *lncRNA4789* and *miR-381-3p* needs to be determined using assays such as competitive binding displacement assay.

According to previous studies, a complex regulatory mechanism involving multiple genes and pathways underlies IMF deposition [3,86,87]. Besides *lncRNA4789/miR-381-3p/FABP3*, we also identified other DE mRNAs between high and low IMF, such as ANGPTL4 [88], CPT1A [89], OLR1 [90], PCK1 [91], and KLF5 [92] that participated in fat deposition via the *PPAR* signalling pathways, adipogenesis and fat cell differentiation (Supplementary File Table S9). Although excluded from ceRNA construction, these were differentially expressed between low and high IMF in longissimus dorsi tissues, suggesting these genes may exert a synergistic effect on IMF deposition via other regulatory mechanisms. In addition, activated *PPAR* signalling may promote adipogenesis through the upregulation of downstream transcription factors, such as *FABP3* in our study which may subsequently enhance the transcription of its target genes, *PPARγ* and *C/EBPα* [11,93]. The persistent inhibition of *FABP3* with its siRNA, *miR-381-3p* mimics or *lncRNA4789* inhibitor was also observed to almost completely suppress adipocyte differentiation and lipogenesis (Figure 6A,B). Moreover, in the *PPAR* signalling pathway, we detected that the expression of multiple genes involved *FABP3* in *miR-381-3p* mimics and *lncRNA4789* inhibitor groups (Figure 6C,D), which further revealed an interaction network of these genes in adipocyte cells, based on previous reports [88,93] (Figure 7). Our findings not only revealed the importance of *FABP3* in IMF deposition but also the role played by multi-gene co-regulation which is exerted via participation of the *PPAR* signalling pathway in IMF deposition. However, further research on molecular communication among the multiple genes involved in the co-regulation of IMF deposition is needed.

## 5. Conclusions

In this study, whole-transcriptome sequencing was performed between low- and high-IMF samples from the longissimus dorsi muscle of Ber × AQSW crossbred pigs, where we screened DE mRNAs, miRNAs and lncRNAs. The DERNAs displayed WGCNA and ceRNA regulatory mechanisms which indicated that the *miR-381-3p*/*FABP3*/*lncRNA4789* system was involved in IMF deposition. At the cellular level, *FABP3* promotes preAD differentiation and lipid droplet deposition in the *PPAR* signalling pathway via a ceRNA regulation mechanism (*miR-381-3p*/*FABP3*/*lncRNA4789*). Our research will aid in the future exploration of the hitherto unrecognised role played by the ceRNA network in Ber × AQSW crossbred pigs in IMF deposition. In addition, this study also identified novel molecular markers for the optimisation of IMF, which is of important breeding and market economic value for improving pork quality.

## Figures and Tables

**Table 1:** Phenotype measure in pork quality and carcass traits for 52 crossbred pigs (Berkshire × AQSW).

Traits	Germs	*n*	Max	Min	Mean	SD
Pork quality traits	IMF (%)	52	6.6	1.4	3.885	1.313
	24 h Drip loss (%)	52	4.549	0.500	1.870	1.180
	Marbling Score	52	4.500	3.000	3.640	0.394
	Total Protein (%)	52	26.58	19.17	23.269	1.528
	45 min Meat Colour (L value)	52	39.203	31.963	35.588	1.839
	24 h Meat Colour (L value)	52	51.440	35.550	41.993	3.857
	45 min pH value	52	6.803	5.520	6.137	0.352
	24 h pH value	52	6.300	5.407	5.673	0.175
Carcass traits	Live Weight (kg)	52	112.500	85.150	102.060	6.622
	Carcass Weight (kg)	52	42.200	29.800	37.796	2.710
	Average Backfat (mm)	52	97.560	24.790	39.374	11.101
	10th/11th Ribs Backfat (mm)	52	89.910	17.900	34.144	11.521
	Carcass Length (cm)	52	110.000	85.000	98.670	5.957
	Carcass StrL (cm)	52	94.000	72.000	85.230	4.764
	Carcass DiaL (cm)	52	92.000	64.000	82.490	6.089
	Loin eye area (cm^2^)	52	32.300	69.200	49.022	5.724

**Table 2:** Differentially expressed mRNAs, miRNAs, and lncRNAs between low and high intramuscular fat in crossbred pigs (Berkshire × AQSW).

Expression	mRNA	lncRNA	miRNA
Upregulated	668	243	100
Downregulated	213	151	58
Total	881	394	158

**Table 3:** Comparison of pork quality traits in multiple pig populations and breeds.

Population	Breeds	Intramuscular Fat (%)	24 h Drip Loss (%)	Meat Colour _45 min_ (L Value)	BFT (mm)	Loin Eye Area (cm^2^)	Reference
CHIPs	LC	*NA*	*NA*	*NA*	34.0 ± 0.68	41.9 ± 0.95	[65]
	LL	*NA*	*NA*	*NA*	38.1 ± 0.62	38.3 ± 0.86	[65]
	SX	*NA*	*NA*	*NA*	31.0 ± 0.21	48.2 ± 0.30	[65]
	JX	5.18 ± 0.13	1.974 ± 1.7	42.82 ± 2.30	25 ± 8.3	*NA*	[53]
	BMX	2.9 ± 0.011	*NA*	*NA*	39.07 ± 7.02	*NA*	[51]
	ERH	3.1 ± 0.02	1.018 ± 0.91	*NA*	35.17 ± 8.60	30.613 ± 4.79	[51]
WECPs	D	2.54 ± 0.29	*NA*	*NA*	*NA*	*NA*	[58]
	D	2.53 ± 0.27	*NA*	*NA*	*NA*	*NA*	[66]
	Y	1.62–2.94	*NA*	*NA*	*NA*	*NA*	[59]
	D	2.24 ± 0.89	*NA*	*NA*	12.22 ± 2.47	*NA*	[52]
Crossbred pigs	B × AQSW	3.885 ± 1.31	1.870 ± 1.18	35.588 ± 1.840	39.374 ± 11.10	49.022 ± 5.72	Our study
	TB × BMM	3.39	*NA*	47.29	34.56	9.60	[56]
	MS × D	5.45 ± 1.95	*NA*	*NA*	*NA*	17.68 ± 2.68	[60]
	SX × LL	*NA*	*NA*	*NA*	34.7 ± 0.35	47.0 ± 0.48	[65]
	D × SX	*NA*	*NA*	*NA*	29.8 ± 0.54	48.6 ± 0.74	[65]
	D × ERH	2.1 ± 1.0	*NA*	38.5 ± 2.4	*NA*	39.8 ± 6.1	[57]
	DDB × JX	4.06 ± 0.17	1.423 ± 1.3	40.64 ± 2.79	32.3 ± 5.7	*NA*	[53]
	D × L	2.71 ± 0.93	*NA*	45.30 ± 3.10	22.62 ± 4.88	*NA*	[67]

*NA*, no reported in this reserach.

## Data Availability

The datasets presented in this study have been submitted in online repositories, China national center for bioinformation. The website of the repository/repositories and accession number(s) can be found below: https://ngdc.cncb.ac.cn/, CRA007916 (accessed on 1 September 2022).

## Supplementary Materials

The following supporting information can be downloaded at: https://www.mdpi.com/article/10.3390/biology11101497/s1, Table S1 Primers used for the quantitative real-time PCR analysis; Table S2 Summary of phenotype measure in 16 essential amino acids for 52 crossbred pigs (Berkshire × AQSW); Table S3 Summary of mRNA and lncRNA sequencing for each sample; Table S4 Summary of miRNA sequencing for each sample; Table S5 Differentially expression mRNAs, miRNAs and lncRNAs between low and high IMF in crossbred pigs (Berkshire × AQSW); Table S6 Top 20 GO terms of GO enrichment analysis for differentially expression mRNAs; Table S7 Top 20 terms of KEGG enrichment analysis for differentially expression mRNAs; Table S8 Summary of 116 overlapped genes between differentially expression mRNAs and target genes of ncRNAs (miRNAs/lncRNAs); Table S9 GO and KEGG enrichment analysis for 116 overlapped differentially expression genes; Table S10 Summary of differentially expression mRNAs and ncRNAs (miRNAs/lncRNAs) in 34 ceRNAs; Figure S1 Principal component analysis plot for the 12 whole-transcriptome sequencing samples between high and low IMF; Figure S2 Correlation and heatmap plots for mRNAs, miRNAs and lncRNAs between high and low IMF samples in pig longissimus dorsi tissues; Figure S3 Validation of mRNA, miRNA and lncRNA differential expression results between high and low IMF in pig longissimus dorsi tissues; Figure S4 ncRNA target genes screening and GO and KEGG enrichment analysis; Figure S5 Metascape visualisation of the interactome network formed by all 116 overlapped DE target genes; Figure S6 Weighted gene co-expression network analysis for differential expression mRNA between high and low intramuscular fat (IMF) in pig longissimus dorsi; Figure S7 Quantification of protein expression in *PPARγ* and *C/EBPα* between NC and *FABP3*-siRNA; Figure S8 mRNA expression of five lncRNAs in tissues and cells; Figure S9 mRNA expression of related genes involved in the *PPAR* signalling pathway.

## Abbreviations

IMF	Intramuscular fat
CDKs	cyclin-dependent kinases
CKIs	CDK inhibitors
*PPARγ*	peroxisome proliferator-activated receptor γ
*C/EBPα*	CCAAT/enhancer binding protein
*FABP3*	fatty acid binding protein 3
LPL	lipoprotein lipase
AQSW	Anqin Six White pig
Ber × AQSW	AQSW sows were crossed with Berkshire boars
DE	differentially expressed
preADs	pre-adipocytes
WGCNA	weighted gene co-expression network analysis
ceRNA	competing endogenous RNA
WECPs	Western commercial pigs
CHIPs	Chinese indigenous pigs
