# Supplementary material for: miR-381-3p Inhibits Intramuscular Fat Deposition through Targeting FABP3 by ceRNA Regulatory Network
Source: Biology (Basel). 2022 Oct 13;11(10):1497. doi: 10.3390/biology11101497 (PMC9598794; doi:10.3390/biology11101497)
Supplement: Supplementary file 1 [file biology-11-01497-s001.zip › supp figures.pdf]

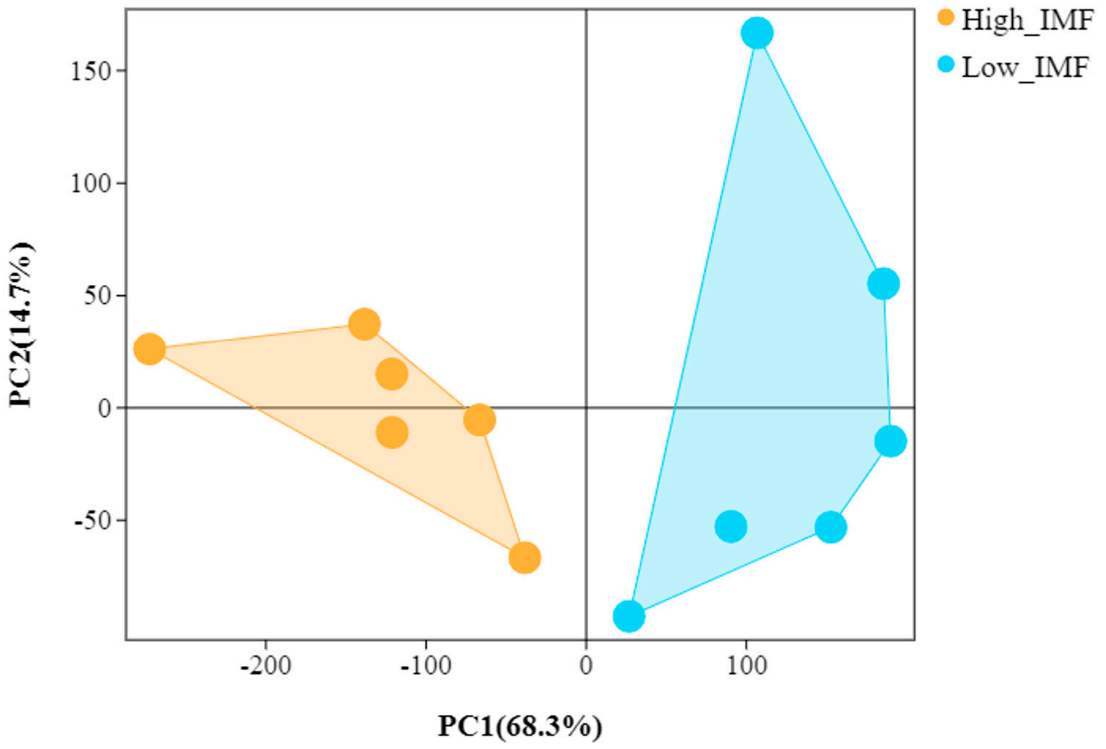

Figure S1. Principal component analysis plot for the 12 whole-transcriptome sequencing samples between high and low IMF.

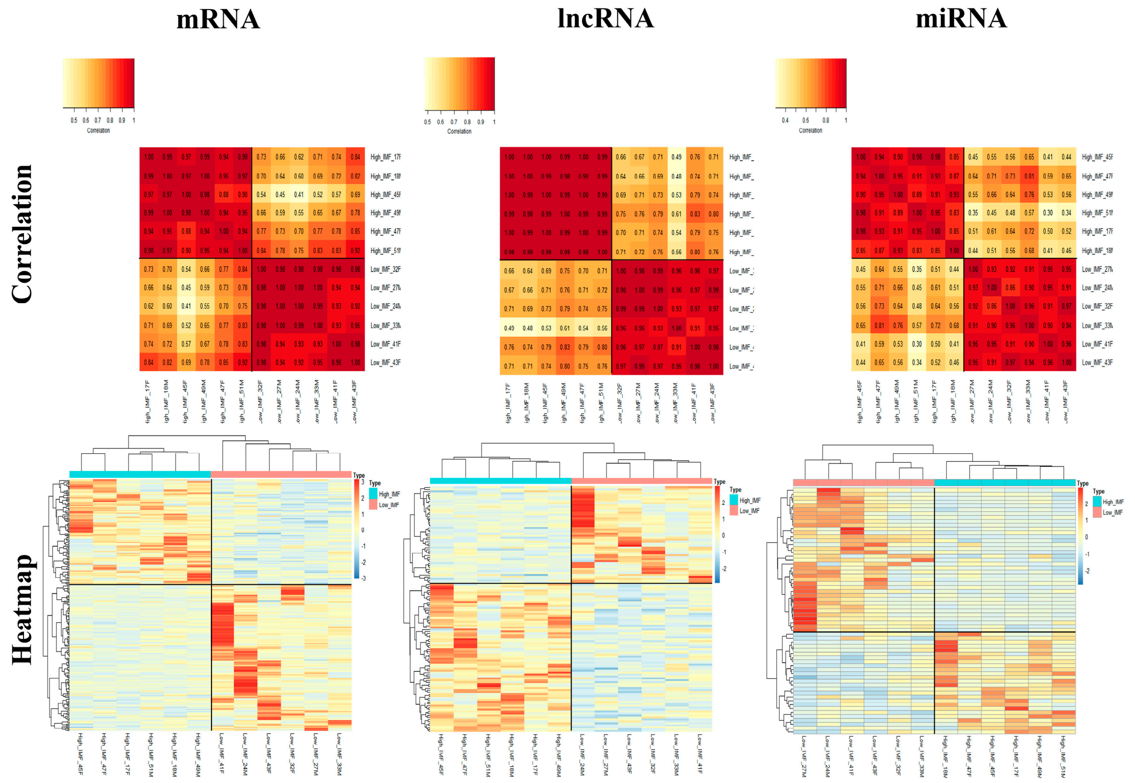

Figure S2. Correlation and heatmap plots for mRNAs, miRNAs and lncRNAs between high and low IMF samples in pig longissimus dorsi tissues.

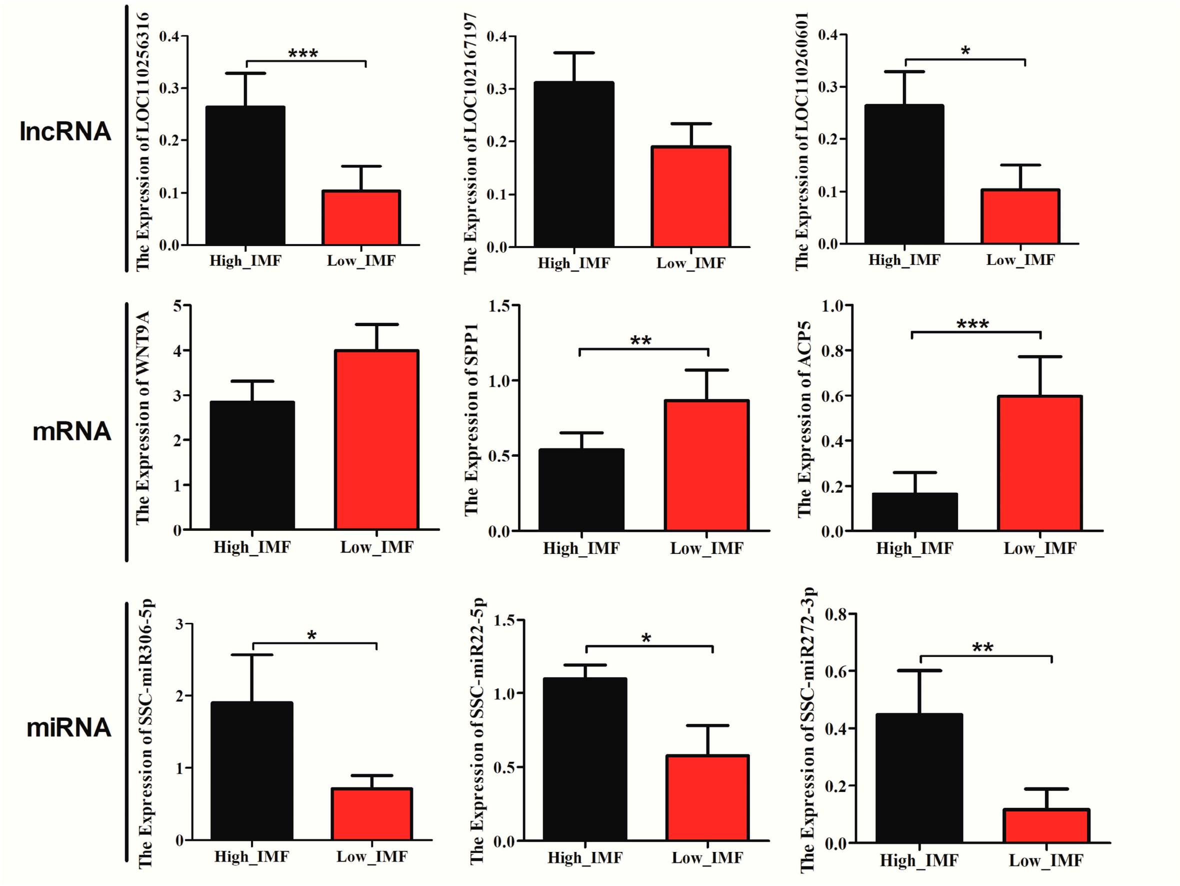

Figure S3. Validation of mRNA, miRNA and lncRNA differential expression results between high and low IMF in pig longissimus dorsi tissues. Three DE mRNAs, miRNAs and lncRNAs were selected randomly and mRNA expression were detected in pig longissimus dorsi tissues. Error bars indicate the mean ± SD of triplicate experiments; * $p < 0.05$; ** $p < 0.01$, *** $p < 0.001$.

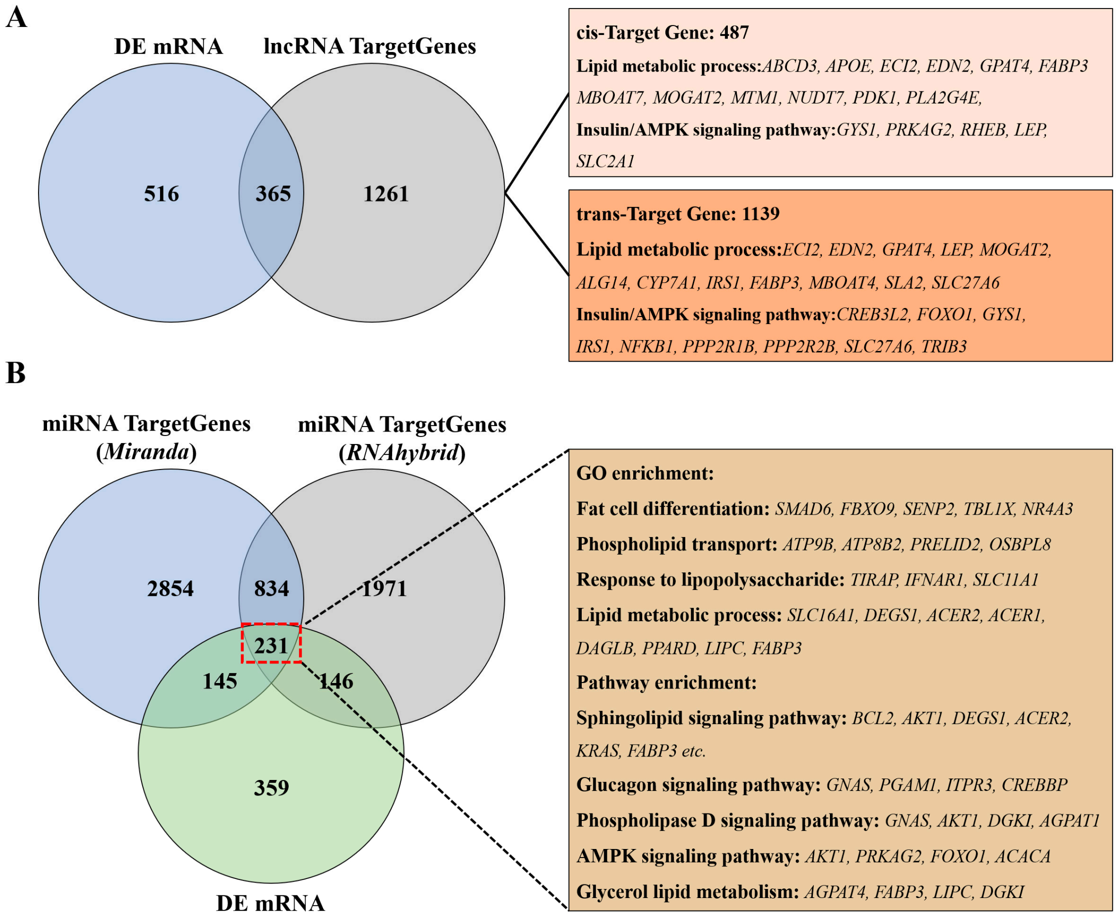

Figure S4. ncRNA target genes screening and GO and KEGG enrichment analysis. (A) Venn diagram for 365 overlapped genes between 881 DE mRNA and 1626 DE lncRNA target genes. (B) Venn diagram for 231 overlapped genes between DE miRNA target genes and 881 DE mRNA.

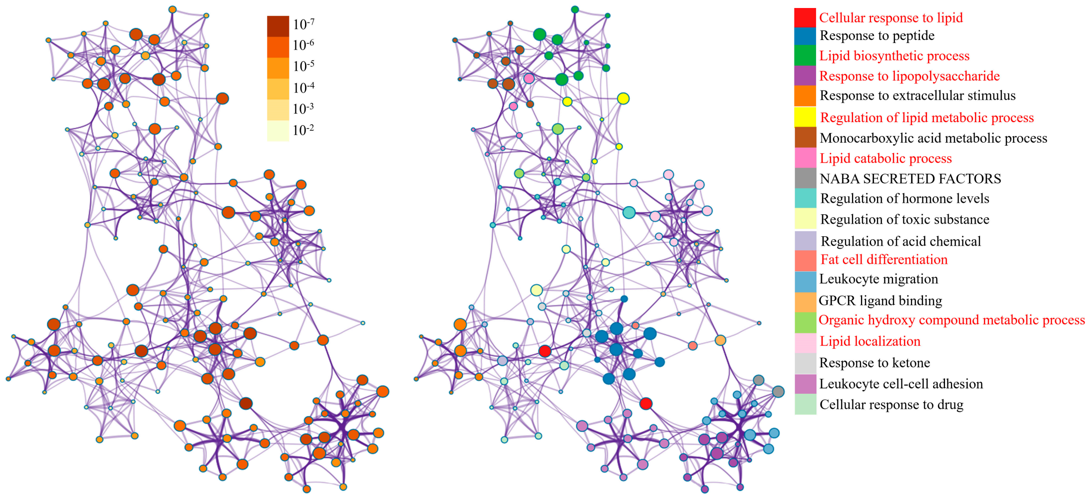

Figure S5. Metascape visualisation of the interactome network formed by all 116 overlapped DE target genes. Left panel: Metascape enrichment network visualisation showing the *p*-value of each enriched term between the intra-cluster and inter-cluster. Right panel: Metascape enrichment network visualisation showing the intra-cluster and inter-cluster similarities of enriched terms, up to 20 terms per cluster. Cluster annotations are shown in colour code. Red text represents the enriched terms associated with lipid-related biological process.

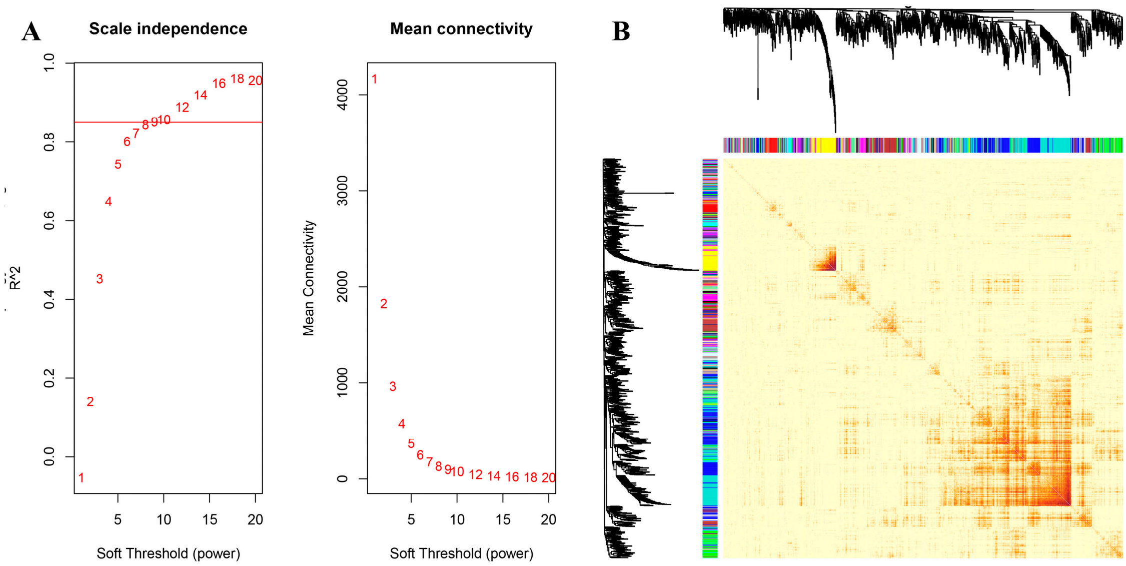

Figure S6. Weighted gene co-expression network analysis for differential expression mRNA between high and low intramuscular fat (IMF) in pig longissimus dorsi. (A) Analysis of network topology for various soft-thresholding powers in high and low IMF. The left panel shows the scale-free fit index (y-axis) as a function of the soft-thresholding power (x-axis). The right panel displays the mean connectivity (degree, y-axis) as a function of the soft-thresholding power (x-axis). (B) Visualising the gene network using a heatmap plot in high and low IMF. Light colour represents low overlap and progressively darker red colour represents higher overlap. Blocks of darker colours along the diagonal are the modules. The gene dendrogram and module assignment are also shown along the left side and the top.

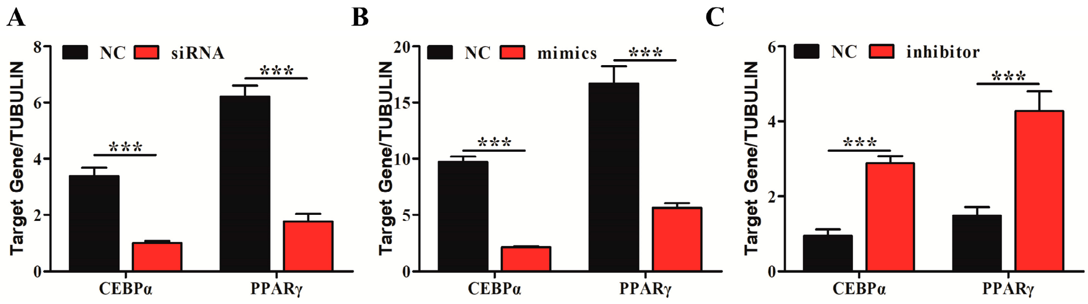

Figure S7. Quantification of protein expression in PPARγ and C/EBPα between NC and FABP3-siRNA (A), miR-381-3p mimics (B) and inhibitor (C). Error bars indicate the mean ± SD of triplicate experiments; *** $p < 0.001$.

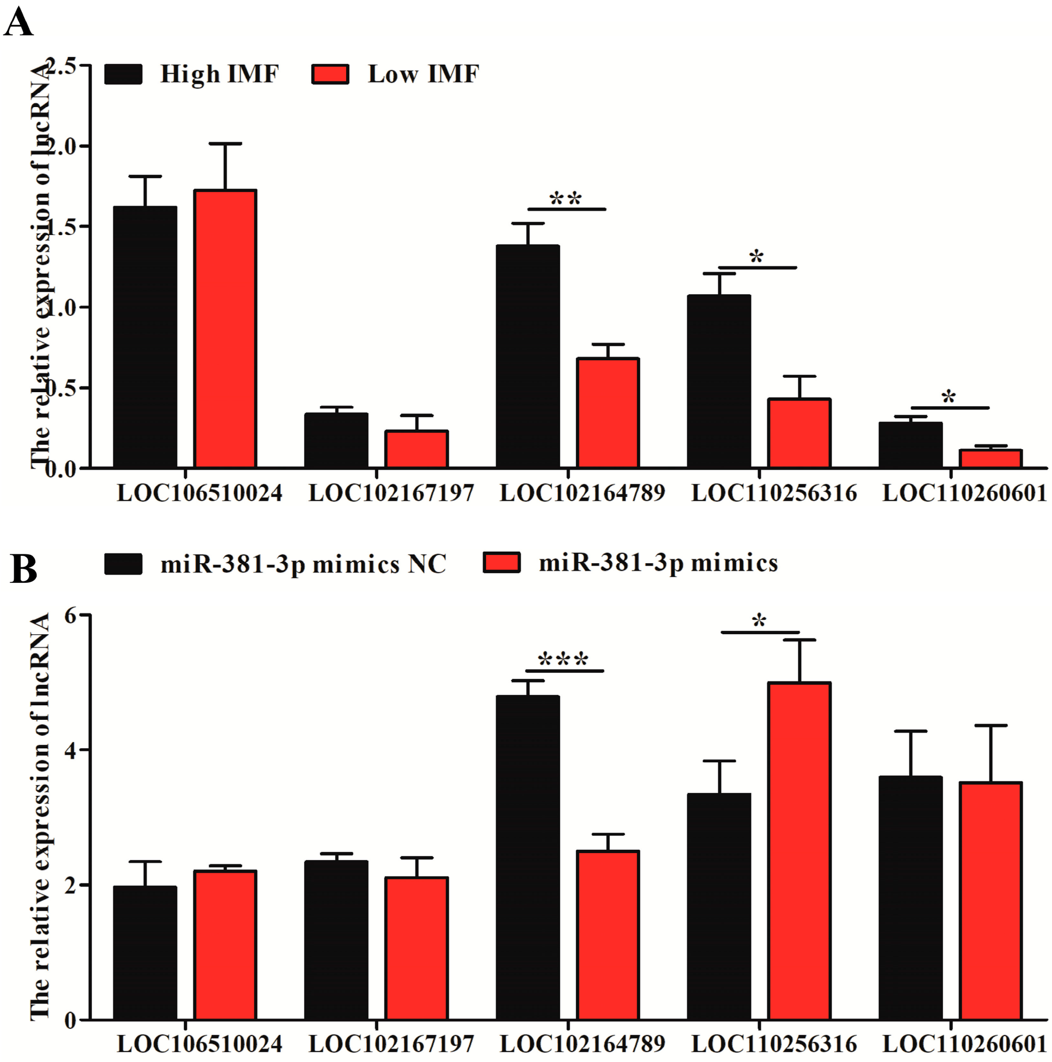

Figure S8. mRNA expression of five lncRNAs in tissues and cells. (A) The mRNA expression of five lncRNAs between high and low IMF in pig longissimus dorsi; (B) mRNA expression of five lncRNAs between high and low IMF in pig adipocyte cell; Error bars indicate the mean ± SD of triplicate experiments; * $p < 0.05$, ** $p < 0.01$, *** $p < 0.001$.

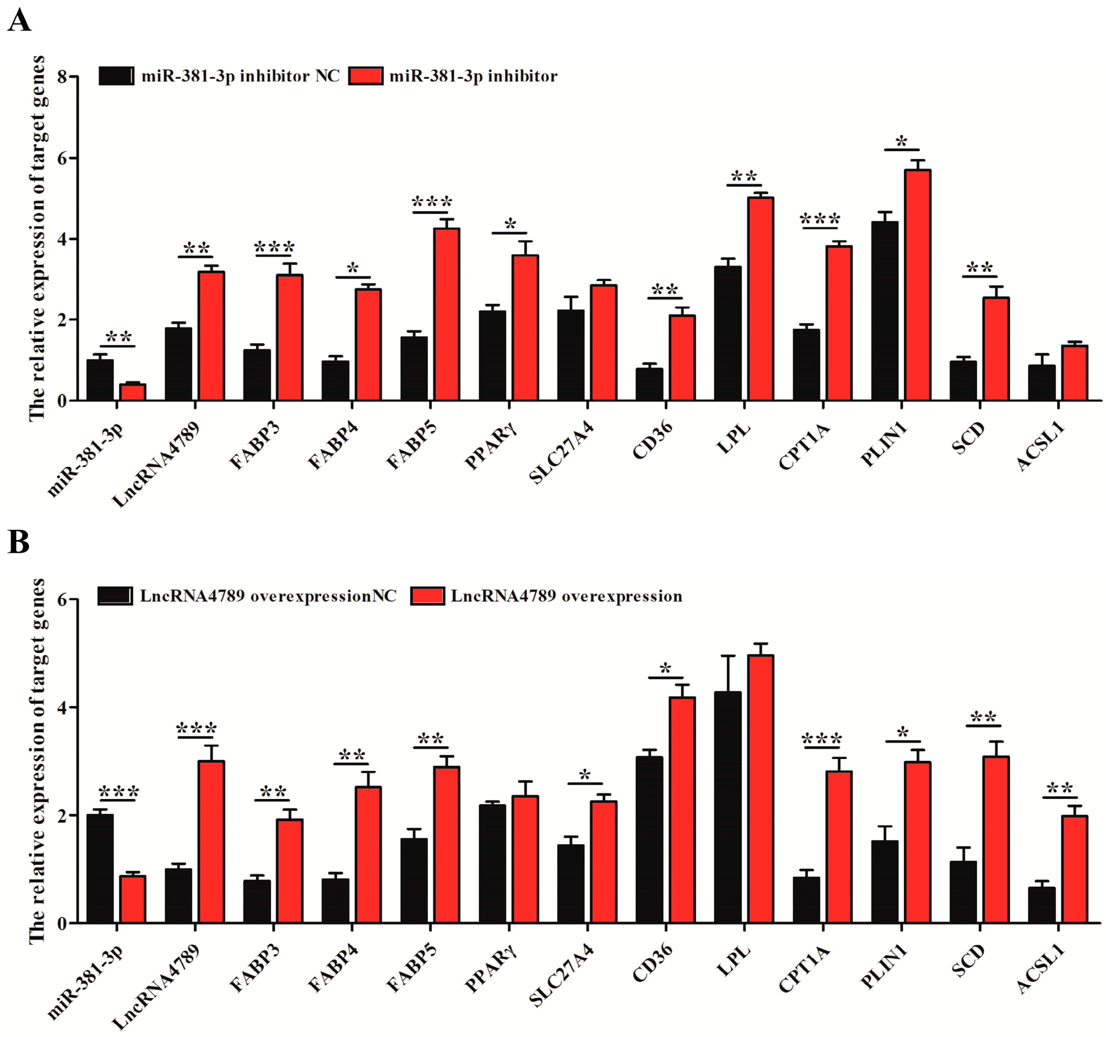

Figure S9. mRNA expression of related genes involved in the PPAR signalling pathway. (A) The mRNA expression of related genes involved in the PPAR signalling pathway, following *miR-381-3p* inhibitor; (B) mRNA expression of related genes involved in the PPAR signalling pathway following transfection with a lncRNA4789 overexpression; * $p < 0.05$, ** $p < 0.01$, *** $p < 0.001$.
